# Guiding the Gaze: How Bionic Reading Influences Eye Movements

**DOI:** 10.3390/jemr18050049

**Published:** 2025-10-01

**Authors:** T. R. Beelders

**Affiliations:** Department of Computer Science and Informatics, University of the Free State, Bloemfontein 9301, South Africa; beelderstr@ufs.ac.za

**Keywords:** bionic reading, eye gaze, reading difficulty, reading

## Abstract

In recent years, Bionic reading has been introduced as a means to combat superficial reading and low comprehension rates. This paper investigates eye movements between participants who read a passage in standard font and an additional Bionic font passage. It was found that Bionic font does not significantly change eye movements when reading. Fixation durations, number of fixations and reading speeds were not significantly different between the two formats. Furthermore, fixations were spread throughout the word and not only on leading characters, even when using Bionic font; hence, participants were not able to “auto-complete” the words. Additionally, Bionic font did not facilitate easier processing of low-frequency or unfamiliar words. Overall, it would appear that Bionic font, in the short term, does not affect reading. Further investigation is needed to determine whether a long-term intervention with Bionic font is more meaningful than standard interventions.

## 1. Introduction

### 1.1. Research Area

**Figure 1:**
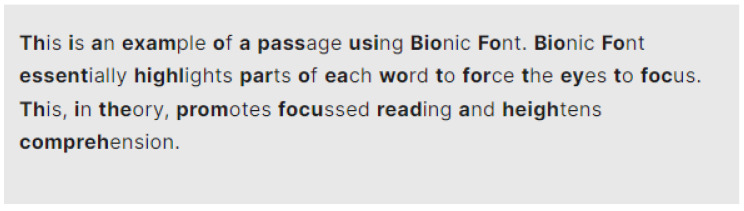
A passage using Bionic font as generated on the Bionic reading website.

Bionic reading is aimed at harnessing the power of technology to aid the reading process. Essentially, Bionic reading, facilitated through the use of Bionic font (Figure 1), adjusts a text so that parts of the words are highlighted or made bold. The idea is that the eyes focus only on these artificial fixation points, thereby allowing the brain to complete the word. Since the brain “reads” faster than human eyes, this technique should foster higher reading efficiency, whilst simultaneously encouraging improved comprehension of the content being read [1].

### 1.2. Research Problem

As synthesised neatly in [2], reading is a fundamental skill that not only empowers individuals to learn about other cultures, societies and subjects but is a prerequisite to be able to function and contribute constructively to society. According to this paper, poor readers with low motivation struggle to learn effectively. Unfortunately, the activity of “reading for pleasure” is on the decline amongst adolescents, and the fear is that poor readers will lead to even more poor readers in future generations, causing a downward slide in societal literacy.

In a world where people are bombarded by content and information, it is further surmised that this has led all ages to become shallow readers of online content, and the Bionic reading tool aims to improve comprehension and assist readers in focusing on the text at hand. Furthermore, a proportion of the population suffers from reading difficulties or attention problems, and Bionic reading is a potential solution that can be used to focus attention and negate some of the problems associated with reading difficulties.

In South Africa, the problem is even further exacerbated by the literacy levels of school-going learners, as tested in 2021. Placed last out of 57 countries, it was found that 81% of Grade 4 learners are unable to read with comprehension in any language, an increase of 3% since 2016 [3]. A previous study [4] on eye movements while reading showed that barkers, readers who decode the text without comprehension, do not exhibit significantly different eye movements from comprehending readers, but that barking is not characterised by the same eye movements as mindless reading or mind-wandering. Hence, barking, or reading with superficial or no comprehension, is an entirely separate phenomenon and not linked to mindless reading. This study could unfortunately not base an intervention on the findings, as more investigation into the phenomenon of barking is required.

### 1.3. Research Objective

Bionic reading is perhaps a method that can be used in such scenarios to encourage readers to learn to focus and move the eye to correct fixation points in order to read. It can thus be investigated whether struggling readers can be taught how to focus more efficiently and effectively while reading, thus improving both reading and comprehension. If Bionic reading is a viable solution for barking and low-comprehension readers, the literacy crisis in South Africa could be on the cusp of a solution.

The objective of this study is to investigate potential solutions and aids that can be employed to alleviate the reading crisis in South Africa. However, before undertaking such a wide-sweeping study, it must first be investigated whether Bionic reading does in fact lead to improved reading and comprehension for skilled readers, or at least readers who are able to read with comprehension.

### 1.4. Research Aim and Question

This study aims to determine how the eye movements of readers are affected by Bionic reading.

The research questions are as follows:

RQ1: In what manner does Bionic font affect the gaze behaviour and eye movements of readers?

RQ2: To what extent does Bionic font affect the gaze behaviour and eye movements of readers?

These answers to these questions will be provided in this paper.

## 2. Background

### 2.1. Eye Movements While Reading

Saccades and fixations are basic eye movements that are used to, respectively, position the eye on an object of interest and then “see” or process that object [5]. Saccades are high-velocity, ballistic movements of the eye, while fixations are the periods of time that the eye is held relatively still, and depending on the task at hand, the length of the fixations can differ [5].

Saccades and fixations are of interest when reading, as their behaviour can indicate whether a reader is experiencing difficulty with the text at hand [5]. The length of a typical fixation when adults read English text is 200–250 ms, and a saccade generally spans 7–9 letters [5]. When a reader experiences difficulty in reading, the length and number of fixations generally increase [6]. Saccades can be either in the forward or backward direction. Forward direction advances the eye to the next piece of text to be read. Backward regressions can be line sweeps or regressions. Line sweeps, while also regressive, are longer and are used to move the eye to the start of the next line of text. Shorter regressions, such as in-word regressions, indicate difficulty in reading. Proficient readers are more adept at positioning their eyes accurately with regressions and thus have fewer regressions than weak readers [5]. All these measures and movements provide a robust method of determining how a reader is performing and what poses difficulties to a reader while engaged in reading of any sort. Therefore, eye tracking was used in this study to capture the gaze of readers in order to answer the research questions.

### 2.2. Effects of Altered Styling

Smaller font sizes cause an increase in fixation duration while reading, as does the use of the sans serif font. While these results were not significant, they indicate that how the text is presented can have an effect on reading [7]. For instance, reading text with rounded letters is faster than when angular letters are used [8]. Letter width also affects reading behaviour, as fonts with condensed letter width result in fewer fixations and saccades and longer fixations, but reading speed was not affected as readers adjusted to the font [9].

Readers with visual impairments may be more likely to struggle reading a presented text. In fact, changing the contrast between the text and the background or blurring the text—effectively degrading the viewing conditions—does change reading behaviour, causing readers to experience more difficulty in reading the text [10] and leading to slower reading times and longer and more fixations [11]. This is confirmed by similar studies that have shown changing text spacing or adding symbols as spaces between words affects reading, particularly in older readers [12], while these readers also struggle more when the inter-letter spacing is condensed [13]. This does not extend to younger readers, even weak or dyslexic readers, where increased spacing between letters reduced errors but did not increase reading speed [14]. What must be kept in consideration is that as font size increases, so too do the number of fixations and saccadic angle [15]; therefore, increasing font size might not be the most efficient means of improving reading, even for visually impaired readers. Since changing the way in which text is presented clearly impacts the reading behaviour, both positively and negatively depending on the change, there must be an ideal presentation format that can enhance reading and enable readers to read more skilfully, effectively and efficiently. Bionic font is aimed at focusing the reader and providing a means to assimilate the text with more understanding and depth. Thus, Bionic reading could also present a solution in this regard.

### 2.3. Bionic Reading

As discussed in a previous section, return sweeps are an eye movement naturally present in reading when the eye moves to the start of the next line of the text. Under conditions where the initial word in a line was presented in bold text (similar to the use of Bionic font), line sweeps were more accurate, resulting in fewer corrective saccades to position the eye correctly. However, they did not result in shorter fixation durations or other reading improvements [16]. In contrast, placing a visual cue, such as a diamond at the start of a new line, significantly reduced reading time and the number of leftward saccades and fixations, while other techniques, such as repeating the final word in a line, did not show the same improvement in reading [17]. Skilled readers are more adept at positioning the eye correctly [5], thereby reducing the number of corrective saccades; therefore, these aforementioned additions could allow lower-skilled readers to more quickly learn effective eye placement when making line sweeps.

Low-frequency words presented in bold are recognised faster than non-bold low-frequency words, but this advantage is not extended to include high-frequency words [18]; hence, to some extent, making text bold can potentially improve reading. Readers with central vision loss are also not assisted by presenting text in bold, as the reading speed does not improve [19] and may actually be negatively affected by the use of bold text [20]. These results further illustrate that the use of bold text, such as with Bionic font, can serve to increase reading proficiency under some circumstances, but that use thereof must be carefully evaluated, as it could also have a negative impact.

Snell [21] conducted a study comparing reading when using regular text to using text formatted with Bionic font. Thirty-two participants read 50 normal text passages and 50 Bionic font passages. Passages were from Dutch news websites, and passages were, on average, 45 words long. The presentation of the passages was counterbalanced, and the passages were occasionally interspersed with questions about the previous text. No difference in reading speed was detected [21], either for the entire trial or the latter half, when participants were more familiar with Bionic font and might have adjusted well enough to read faster. Additionally, when dividing readers into slow and fast readers, there was also no difference in reading speeds for either of these groups. It was thus concluded that Bionic font does not improve reading. Since Bionic font is aimed at counteracting superficial reading and increasing comprehension, it could perhaps benefit readers who are not neurotypical. However, EEG results suggest that Bionic font does not offer any added benefit to ADHD readers [22]. For younger second language readers, reading proficiency (the ability to read, comprehend, interpret and decode text) was greatly improved when an intervention using Bionic font was introduced [23]. These chosen readers (*n* = 30) were initially struggling as they could not understand the texts they had to read, had limited vocabulary and had difficulty understanding grammatical structure [23]. A teaching intervention using Bionic font was introduced and presented to these students. Pre- and post-tests after the Bionic font intervention showed a significant improvement overall in reading proficiency [23]. In a similar study, it was found that reading comprehension was not improved through the use of Bionic font [24]. Nevertheless, the use of Bionic font increased the reading motivation and self-efficacy of students with learning disabilities [25].

Hence, poor readers are more likely to benefit from the use of Bionic font to increase reading proficiency.

## 3. Materials and Methods

An eye-tracker allows eye movements to be captured while participants look at a presented stimulus [26]. A remote eye-tracker is non-invasive and is generally mounted below a normal computer screen. The participant is therefore not hindered in any way and is not aware of the eye-tracker at all. For the purposes of this study, a Tobii Spectrum eye-tracker (Tobii, Stockholm, Sweden) was used to record data at a frequency of 600 Hz. Texts to be read were presented on a normal computer screen. Participants were seated approximately 60 cm from the screen and were free to move during testing.

Participants were adults, students or staff members at the university where the study was conducted, who were fluent English readers, but not necessarily first language speakers. Hence, the sample was a convenience sample, and only fluent English readers were selected. A pre-test self-assessment questionnaire was administered to ensure that all participants considered themselves fluent in English reading. Questions were posed as to the reading habits of the participants (how often they read), the reading format they employ (digital or hard-copy) and the type of text they read (fiction, non-fiction, etc.). In addition, participants were asked whether they would classify their reading speed as slow, intermediate or fast. These demographics were captured mainly to ensure that all participants were comfortable reading in English, and on-screen reading was not used for this paper to distinguish between participants in any way.

A total of 53 participants were included in the study. Participation was voluntary, and participants were verbally invited to participate. All participants had normal or corrected-to-normal vision. All but 8 participants indicated their primary reading language was English. Of those 8, only 1 did not specify English as an additional reading language. For this participant, it was verbally confirmed that they were comfortable reading in English. The majority of the participants (*n* = 24) were between the ages of 18 and 25, with a further 14 between the ages of 26 and 35, 7 between 36 and 46, and 7 between 45 and 60. One participant declined to indicate their age bracket.

Participants were welcomed to the laboratory, a brief explanation of the study was given, and the consent form was explained. Participants were asked to sign the consent form and complete the pre-test questionnaire next. Thereafter, participants were asked to read two passages each, one in standard text and one using Bionic font. Presentation of passages was counter-balanced to avoid bias due to order precedence. Therefore, some participants read passage 1 first, others passage 2, and furthermore, some read in Bionic font first and others in standard font. Each participant read passage 1 and passage 2, but never in the same font; hence, if passage 1 was read in standard font, then passage 2 was read in Bionic font and vice versa. Participants read silently and at their own speed. Each was followed by a 10-question multiple-choice comprehension test for the passage just read. The duration of the individual sessions was reliant on the participants’ reading speed, with the fastest reading time (total for both passages and excluding time to complete questionnaires) lasting 9 min and 76 s, and the longest 36 min and 37 s. The average reading time was 21 min 40 s (sd = 367 s).

## 4. Ethics Statement

Participation was voluntary, and participants were free to leave at any time if they chose to. The purpose of the study and what was expected of them were explained to each participant before the commencement of the session. All participants signed a written informed consent form prior to the test session. The research was approved by the General Human Research Ethics Committee (GHREC) of the University of the Free State under protocol number UFS-HSD2023/1818 on 5 October 2023, and an extension was granted for the study on 2 September 2024 and then again on 4 September 2025. Hence, it is declared that the investigations were conducted in accordance with the principles outlined in the Declaration of Helsinki (1975, revised in 2013).

## 5. Stimuli

Extracts from the Nobel Laureate Ernest Hemingway’s book The Old Man and the Sea were used as the stimuli. This allegorical book was chosen since Hemingway’s writing style is considered clear, direct and calm, yet very descriptive [27]. A stylometry analysis of the book reveals that the average word length is 3.8 and the average sentence length is 14.4, and the book is considered to be at a fourth- to fifth-grade reading level [28]. While the text does contain longer words and complex sentences, Hemingway strives to make the reader’s task easy and does not seek to confound or burden the reader by expecting them to analyse meaning, preferring instead to make his intention clear [27]. This might seem as though the text was too easy for adult readers, but the enduring nature of the book and the fact that it describes complex themes and relationships place it in a unique category. Additionally, the study did not aim to make the reading overly difficult; hence, a passage that was relatively easy yet challenging and interesting enough for adult readers was chosen, and this book was deemed to be suitable for the purposes of the study.

Two passages were selected for inclusion in the study. The first passage was from the opening of the book and contained 1141 words, with an average word length of 3.9. The difficulty of the passage, determined using the Flesch–Kincaid scale, was 85.6, with a grade level of 5.7. The second passage was taken from further in the book, where the old man has already put out to sea and is fishing as the sun rises. This passage contained 1476 words, with an average length of 4, and was at a difficulty level of 82.8 and a grade level of 6.1. Hence, the two passages were similar in length and difficulty.

**Figure 2:**
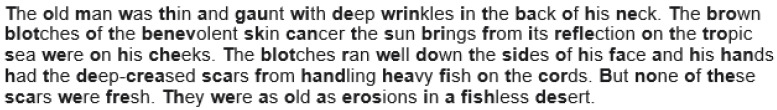
Sample of The Old Man and the Sea using Bionic font.

Each passage was prepared using Arial text and Arial text with Bionic font applied. Using a Latin square, the order of the passages and the font used was counterbalanced to ensure that no learning effects, fatigue or other variables influenced the results. Line spacing was increased to ensure the accuracy of eye-tracking results. A sample of the text, using Bionic font, is shown in Figure 2.

## 6. Results

### 6.1. Reading Speed

The reading speed of each participant was calculated for each passage they read. The average reading speed for the passage in standard font was 131.1 wpm (sd = 39.2) and 130.8 wpm (sd = 37.7) in Bionic font. These speeds are slower than the typical adult English reader, which may be an indication that the sample, in general, did not comprise strong readers, but since for most this is their second language, it is not entirely unexpected and hence not considered a cause for concern. Additionally, analysis was performed pair-wise, so the comparison is within-subjects, and only individual measures are important. On average, however, the reading speed for Bionic reading was slower. A paired *t*-test (pairing each participant’s standard reading speed and Bionic reading speed) showed that the difference in reading speed was not significant (t(52) = 0.134, *p* = 0.9) between the two font styles. Therefore, in terms of reading speed, Bionic font has no significant influence on reading behaviour.

**Figure 3:**
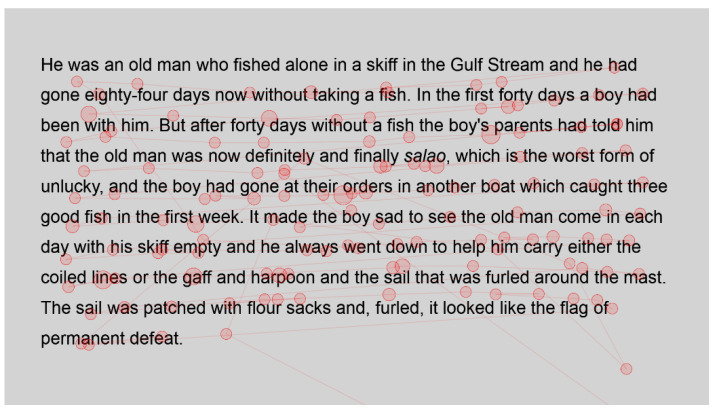
Gaze plot of fastest reader for standard font.

**Figure 4:**
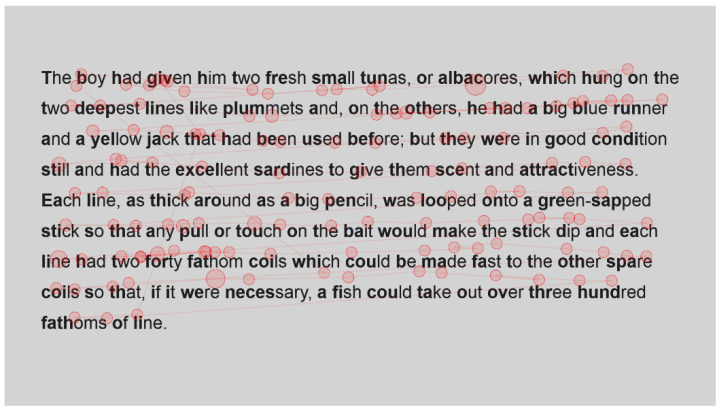
Gaze plot for fastest reader for Bionic font.

**Figure 5:**
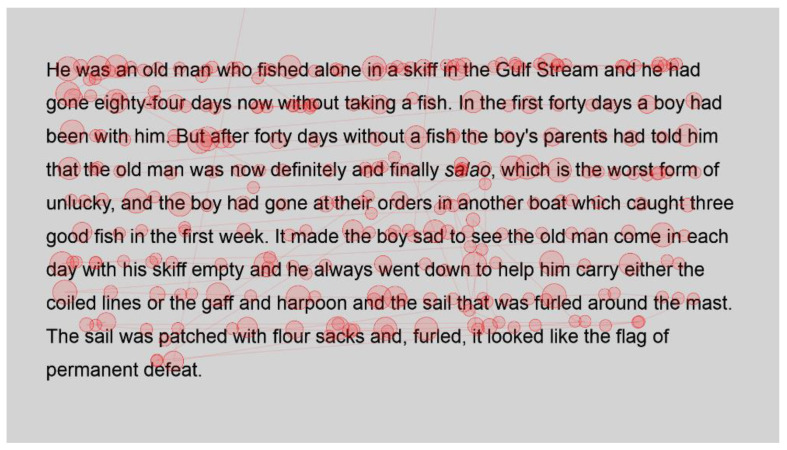
Gaze plot of intermediate reader for standard font.

**Figure 6:**
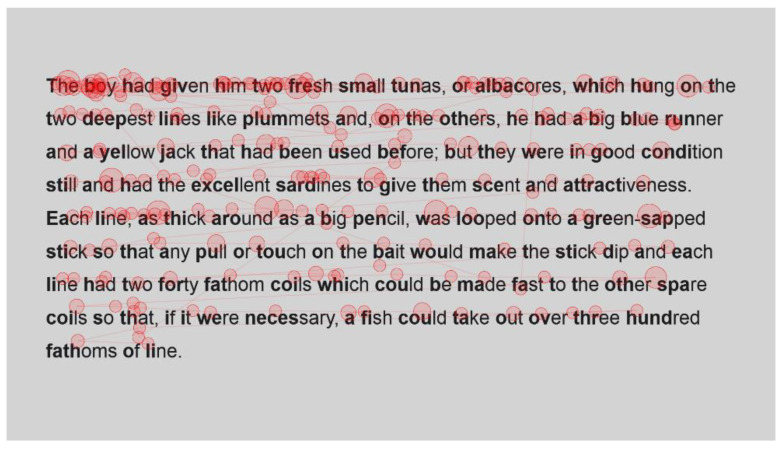
Gaze plot of intermediate reader for Bionic font.

**Figure 7:**
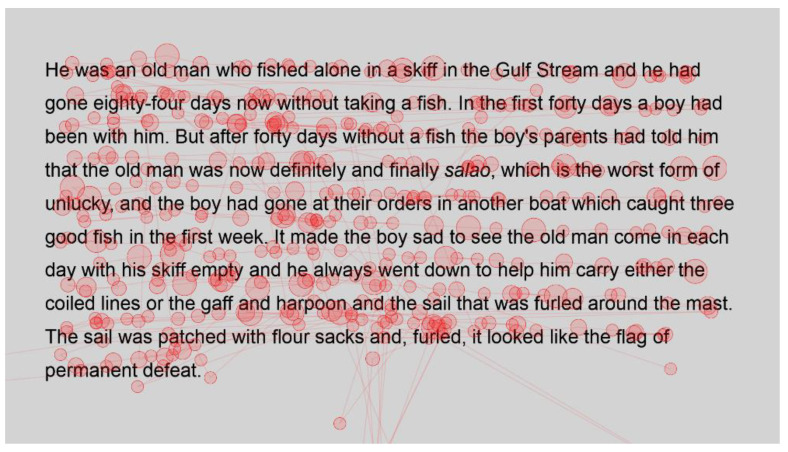
Gaze plot of slowest reader for standard font.

**Figure 8:**
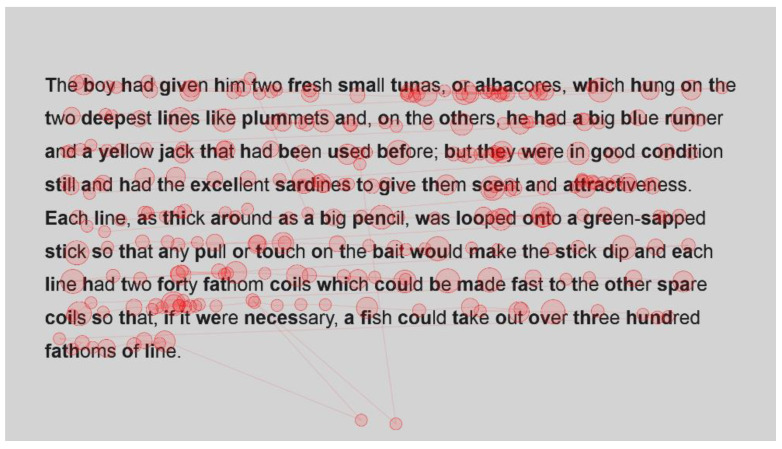
Gaze plot of slowest reader for Bionic font.

For the sake of interest, the gaze plots (Figure 3 and Figure 4) of the fastest reader, a reader who read with an intermediate speed (Figure 5 and Figure 6) and the slowest reader (Figure 7 and Figure 8) were extracted for their first page of standard reading and Bionic reading. These are considered representative of the sample and will be analysed anecdotally. The size of the fixations (shown as circles) is relative to the length of the fixations; therefore, larger dots indicate longer fixations. The lines indicate saccades and connect successive fixations.

The fastest reader (Figure 3 and Figure 4) had comparative fixations throughout their passages, as there do not seem to be fixations with lengthened durations, and the number of fixations appears comparable. Fixations for this reader were also not overly long, showing that the passages were easily read and did not present any difficulties.

Visual comparison of the fastest reader’s gaze plot with the intermediate (Figure 5 and Figure 6) shows an increase in both the number and length of the fixations of the intermediate reader. However, apart from a high cluster of fixations at the start of the Bionic font passage, the spread appears comparable for this reader. It could be that the intermediate reader took a moment to familiarise themselves with Bionic font as the passage was first displayed. Fixation lengths appear slightly longer than the fastest reader, which is to be considered illustrative of the fact that this was a weaker reader, but they do not appear to be overly long either. This reader needed more fixations in order to process words, for example, Gulf and harpoon. This is in contrast to the fastest reader, who had single fixations on most words. In this instance, there are multiple fixations on each word, signifying the need to fixate more or an increase in regressions.

The slowest reader clearly experienced more difficulty with the passages (Figure 7 and Figure 8), as there is a marked increase in the number of fixations throughout the passage. Once again, however, a paired visual comparison indicates that between the standard and Bionic font, there is little difference. Multiple fixations, of approximately equal duration, are present on most words, indicating that the reader was experiencing some difficulty in reading the passage.

Inspection of these gaze plots therefore clearly shows that the number of fixations increases as the reading speed of the reader decreases, which is to be expected. It also illustrates that this is also the case, even when using Bionic font, there is no discernible difference between the eye movements of these readers as the presentation changes. The next section will statistically analyse the number of fixations.

### 6.2. Number of Fixations

The number of fixations gives an indication of how many fixations were needed to read the word and is also elevated for words that needed repeated viewing (regressions). Hence, the more fixations there are, the more the reader struggles with the text [5]. For each participant and passage, the number of fixations was calculated and analysed.

**Figure 9:**
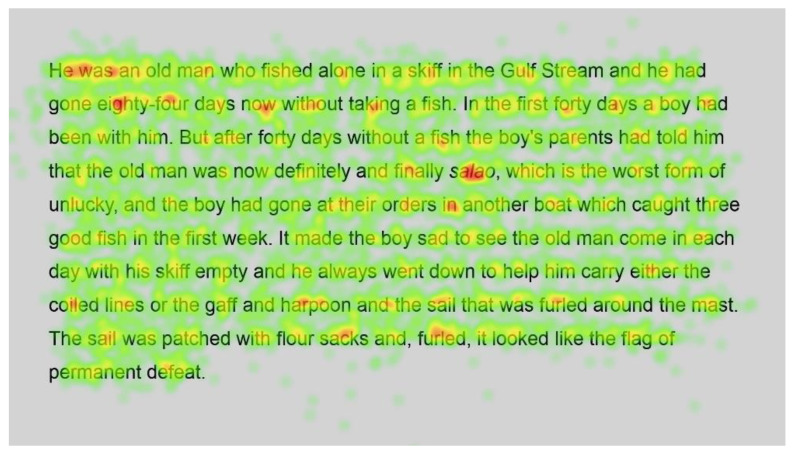
Heatmap of number of fixations (*n*) for passage 1 using standard font.

**Figure 10:**
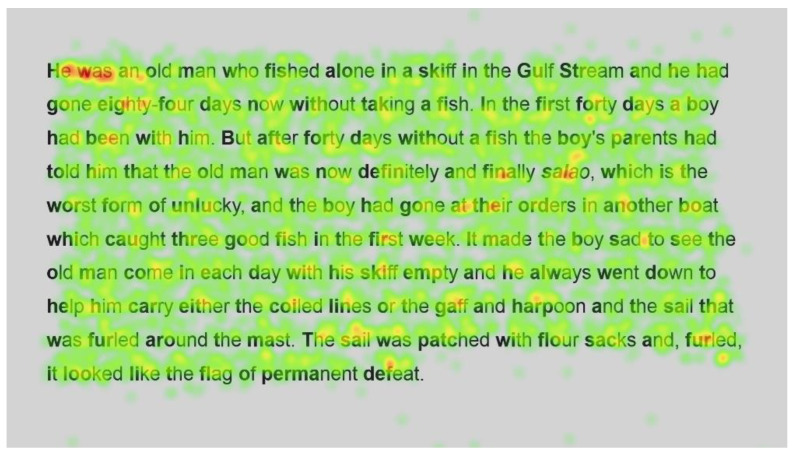
Heatmap of number of fixations (*n*) for passage 1 using Bionic font.

Heatmaps can also be used to visualise the number of fixations, with warmer colours indicating more fixations and cooler colours indicating fewer fixations. Figure 9 shows heatmaps for the number of fixations for all participants for the first page using standard font, and Figure 10 for the same page using Bionic font.

Both passages show more fixations for the first few words of the passage; this could simply be the reader preparing themselves and orienting themselves to start reading on the screen.

Further into the passage, function words, such as and, the and in, and shorter words, such as boy and man, are fixated on with both standard and Bionic font. Longer words that are familiar (such as fished, taking, another and permanent in Figure 9 and Figure 10, last line) received attention on all syllables of the word but seemingly not more fixations than shorter words. Previous research has shown that word length and predictability of words correlated strongly with the practice of skipping words, whereby shorter words and predictable words are inclined to be skipped by readers. However, when these words are fixated on, they have shorter fixations [29]. Hence, the findings are similar to [29] in that there are fixations on longer familiar words, while shorter words, which are always fixated on here, have fewer fixations.

**Figure 11:**
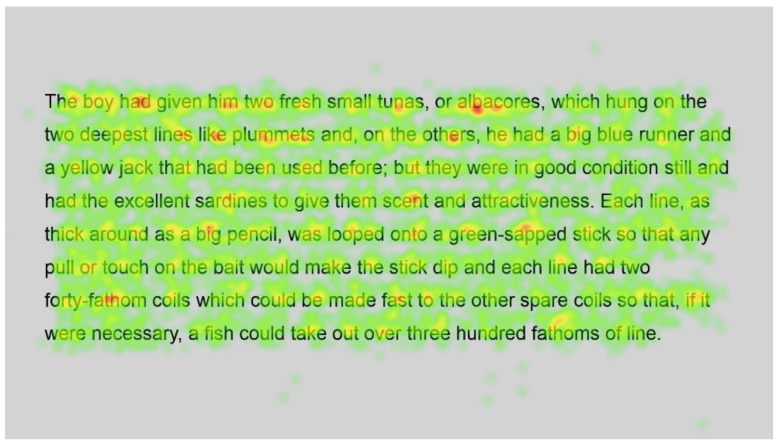
Heatmap of number of fixations (*n*) for passage 2 using standard font.

**Figure 12:**
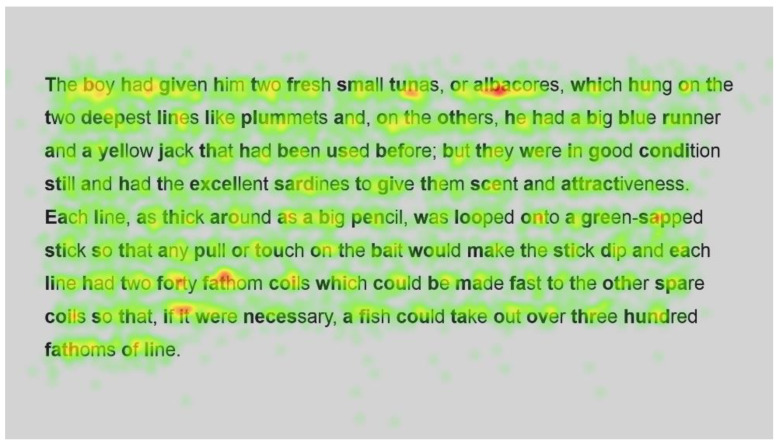
Heatmap of number of fixations (*n*) for passage 2 using Bionic font.

Figure 11 and Figure 12 show the number of fixations for the second passage in standard and Bionic font, respectively. Inspection of these heatmaps shows similar behaviour to passage 1; function words are fixated on but not frequently, and longer words, such as condition, necessary and hundred, are also fixated on but not more than shorter words, with fixations on all syllables.

In contrast, longer words that were situated close to unfamiliar words (such as finally next to the unfamiliar word salao in Figure 9 and Figure 10), or that could have been less familiar and not everyday words (such as albacores and fathom in Figure 11 and Figure 12, line 1), also had a spread of fixations, but are overlaid in red, indicating that there were relatively more fixations on that word. Unfamiliar words, such as salao (Figure 9 and Figure 10, line 4), received more attention in both presentations as they would necessitate more processing to read and make sense of the unfamiliar non-English word and place it in the context of the previously read words. It may also have caused more regressions.

Interestingly, inspection of the heatmaps (Figure 9, Figure 10, Figure 11 and Figure 12) shows that when using Bionic reading, the fixations do not appear to be focused on the start of the word. Rather, it is evident that there are fixations on all parts of the word, and they are largely evenly distributed. The underlying premise of Bionic reading is to focus fixations on certain points—namely the leading letters of the word—and allow the brain to “auto-complete” the word without having to fixate on all parts of the word. This appears, superficially, not to be the case in practice. This applies to all words, including words at the start of a line, so the bold letters do not force a line sweep to land on the leading letters. This phenomenon was seen throughout the passages. Fixation placement will be analysed in more detail in a further section.

Paired analysis confirmed that the difference between the number of fixations per word was not significant (t(52) = 0.30, *p* = 0.8). This further cements the assertion that Bionic reading does not reduce the number of fixations, but that readers still feel the need to execute a similar number of fixations as with standard font. Therefore, it seems unlikely that the reader was able to complete the word just by fixating on the start of the word, since there is no significant difference between the number of fixations required to read the passages.

### 6.3. Fixation Duration

The fixation duration is another measure of reading behaviour, in that longer fixations often show that the reader is experiencing difficulty [5]. Hence, the fixation durations for each participant and each passage were analysed.

**Figure 13:**
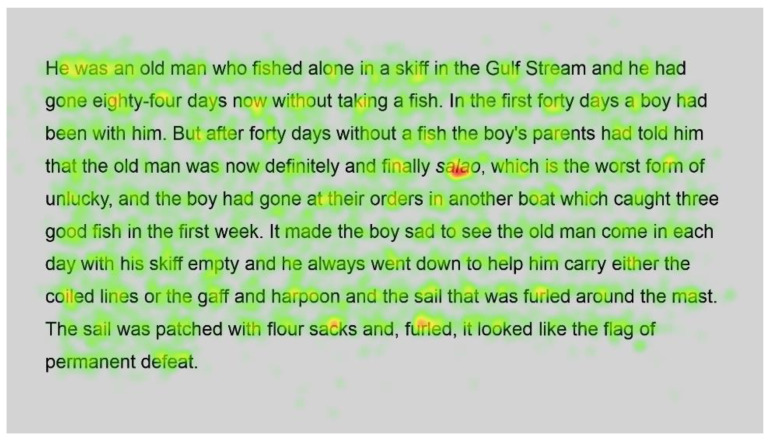
Heatmap showing fixation durations (ms) for passage 1 using standard font.

**Figure 14:**
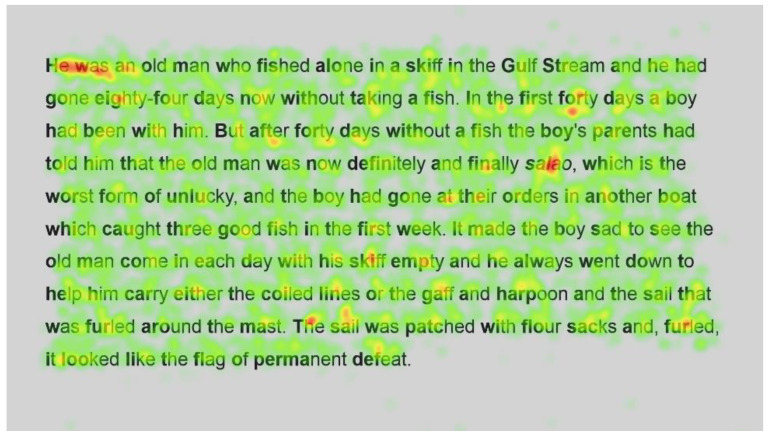
Heatmap of fixation durations (ms) for passage 1 using Bionic font.

Figure 13 and Figure 14 are heatmaps showing fixation durations for the first page of passage 1. As with standard heatmaps, the cooler colours show the shorter fixation durations and warmer colours denote longer fixation durations.

Fixation durations closely mimicked the number of fixations in terms of where there was increased attention and where there was less attention, as could be expected, since the more fixations there are, the longer the total duration would be. Hence, unfamiliar words (salao) were overall fixated on longer, and shorter or familiar words were fixated on for shorter. This naturally correlates with the number of fixations and confirms the findings of [29] in terms of word predictability and length.

As with the number of fixations, there is no discernible or noticeable difference between standard and Bionic font, and the duration of the fixations appears similar across the word.

**Figure 15:**
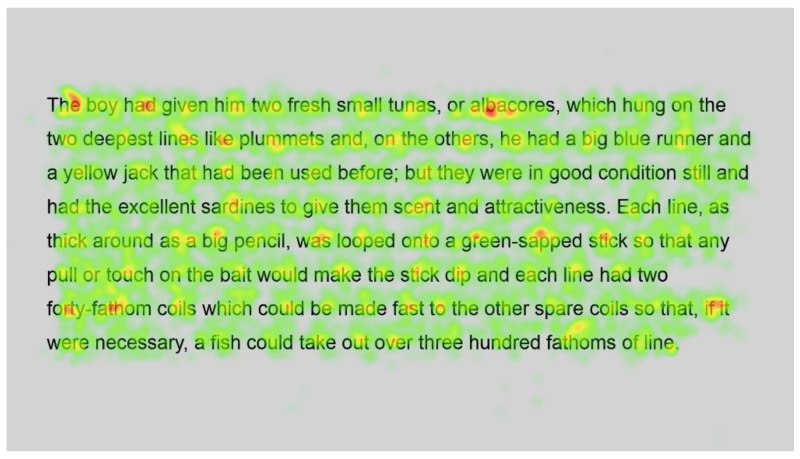
Heatmap of fixation durations (ms) for passage 2 using standard font.

**Figure 16:**
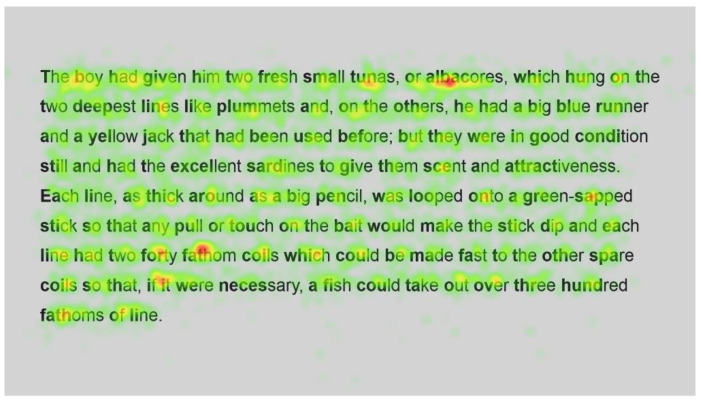
Heatmap of fixation durations (ms) for passage 2 using Bionic font.

Figure 15 and Figure 16 are heatmaps showing fixation durations for the first page of passage 2. The use of the unfamiliar word, albacore, was fixated on longer than sardines further into the passage. The first occurrence of fathom was also fixated on longer than the second occurrence later in the passage; hence, readers were able to remember the word and did not need as long to process the second occurrence as the first.

In the case of Bionic font, the start of the words that are in bold does not elicit longer fixations either. The expectation would be, if Bionic font works as intended, that the leading letters would be fixated on longer and subsequent fixations in the word would be shorter as they would only be needed to confirm the word in the event that the reader was unable to process the word using only the leading letters.

The mean fixation duration when reading in standard font was 232.9 ms and 228.4 ms when using Bionic font. Both these are typical of English fiction reading for adult readers, but Bionic font did have slighter shorter fixations than standard font.

However, this difference in fixation durations was not significant (t(52) = 1.02, *p* = 0.3). Hence, in terms of the duration, Bionic font does not promote more efficient reading than standard font.

### 6.4. Fixation Placement in Word

During typical English reading, fixation placement tends to be to the left of the middle of the word [5]. This allows the reader to read the word optimally using their visual span to “see” the surrounding letters without fixating on them. Heatmaps in prior sections appeared to show that fixations were evenly distributed across all syllables and letters of the word.

For all passages, each word was therefore divided into the first letter, the middle letter, the last letter and, if the length of the word allowed, letters to the left of the middle and letters to the right of the middle. Fixations were then determined for all these positions to determine where the readers fixated. Since Bionic reading is designed to lead the eye, the assumption was that in this instance, the fixations would primarily be placed on the start of the word, contrary to standard font, which should, if following the typical pattern, be largely situated to the left of the word centre.

**Figure 17:**
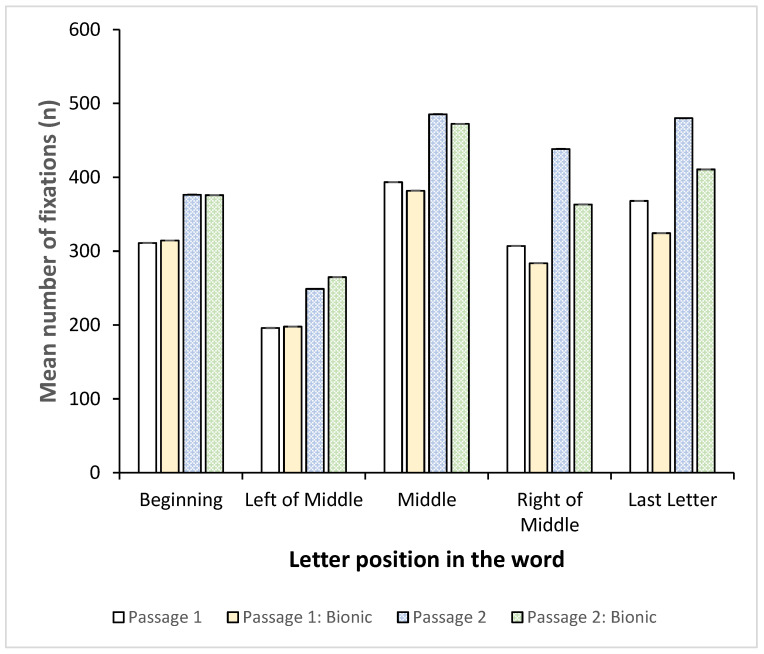
Mean number of fixations (*n*) vs. position of letter in word.

**Figure 18:**
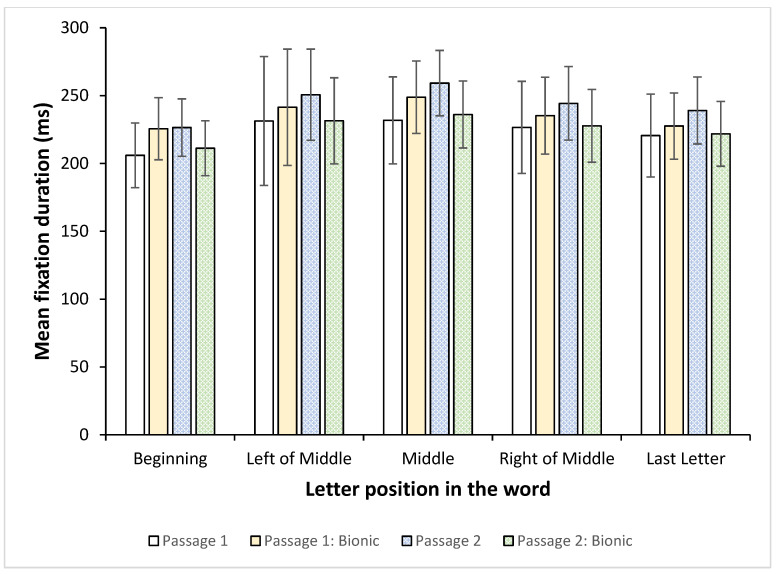
Mean fixation duration (ms) vs. position of letter in word.

Figure 17 and Figure 18 indicate, respectively, the mean number of fixations and mean fixation duration for each letter position in the words for each passage.

Interestingly, with both standard and Bionic reading, the number of fixations was the lowest left of the middle and peaked for all passages in the middle of the word. Even with Bionic reading, the most fixations were in the middle of the word, followed by the last letter. Furthermore, the right of the middle and the last letter also accounted for more fixations than the start of the word. This was evident in both the standard font and the Bionic font. Between the passages, the number of fixations was similar for each letter position; hence, Bionic font does not appear to change reading behaviour and the number of fixations on letter positions.

Figure 18 shows the mean fixation duration for each letter position.

Fixation durations remained approximately the same regardless of the position in the word, showing that the readers did not, on average, spend significantly more time on any particular position.

Hence, while the number of fixations varied based on letter positions, with the most fixations being on the middle and to the right and end of the word, the fixation durations did not vary much. This would indicate that the participants did not require more time to process the words, but that they were not fluent enough to be able to complete the word automatically by fixating on the start(ing) letter(s). Instead, they had to fixate on more letters, and, in particular, the trailing letters, in the word in order to make sense. Since this is present in both standard font and Bionic reading, it appears that the addition of Bionic font does not enhance fixation placement and word completion as expected.

## 7. Discussion

Fixation placement was largely at the discretion of the reader, regardless of the type of font used, and the use of Bionic reading did not force the reader to position their gaze on the leading character. Instead, it appeared that the nature of the text was the dominant factor in this regard, since fixation position was similar for passage 1 and passage 2, regardless of the presentation format. Converse to prior studies, the majority of fixations were in the middle and to the right of the word, but since this was prevalent in both fonts, it appears to be a characteristic of the demographic included in the study, who were not L1 English speakers. Since the number of fixations was similar throughout the word, it can be concluded that the participants were not capable of completing the word, but required more fixations in order to read the letters and were unable to rely on visual span to detect letters either.

Leading letters at the start of a line showed similar behaviour, with the participants not fixating on the start of these words either. Previous studies [17] have shown that a visual aid, at the start of the line, improves the accuracy of line sweeps. Further analysis is required to determine whether the line sweeps were more accurate, but based on the fact that there were no elevated numbers of fixations at the start of the line for either font, it appears that the line sweep was not an influencing factor for this group.

Converse to previous studies [18], low-frequency words still required extended viewing time to be read, and this phenomenon was not altered with the application of Bionic font. Previous studies made the entire word bold, which is perhaps more useful than only certain letters that, in any case, present the same as surrounding words. High-frequency words and short words were all fixated on, which contrasts with the typical reading behaviour [29]. Again, since this was evident in both standard and Bionic font, it might be unique to this demographic, who were not L1 English readers.

These previous studies contained similar elements and factors to the current study, but did not include Bionic font per se as a factor. Studies that included Bionic font are more comparable to the current study.

In contrast to [23], it was found that Bionic reading did not improve reading proficiency, although in the previous study, there was a long-term intervention that was staged, whereas the current study was a once-off exposure to Bionic reading. However, as far as can be ascertained, Bionic reading was the only intervention applied during the previous study. Thus, the improvement in reading could have been as a result of following a more directed reading programme and could perhaps have been just as beneficial using standard font and therefore might not be a direct consequence of Bionic reading. Therefore, a direct comparison between these studies is not conclusive, as the current study did not employ a reading intervention, and the previous study did not include a control group or a different intervention that most likely would have been just as successful.

The study closest to the current study, which investigated paired reading behaviour on short texts and without an intervention [21], indicated that reading speed was not affected by Bionic font. This study confirms those findings in that the use of Bionic font did not significantly improve reading speed, although there was a minor decrease in Bionic reading speed. As a result, the direct application of Bionic font does not improve reading efficiency. It is unclear whether the founders of Bionic font meant for it to be applied as a long-term reading aid that will teach a reader to process text more efficiently, but clearly, for a short-term solution, based on these two studies, it does not improve reading speed. The current study analysis can be expanded to determine whether initial reading speed is an influencing factor when using Bionic font. Therefore, a subsequent paper will discuss the findings relative to reading speeds, similar to the more in-depth analysis of [21]. At this point, however, this paper concurs with the findings of [21] that Bionic font is not a suitable method to increase reading speed.

To summarise, the aim of the research was to determine to what extent Bionic font affects reading behaviour. Research question 1 asked how Bionic font influences reading behaviour, and research question 2 asked to what extent the effect is seen. Analysis of the data indicated that there was a minor decrease in reading speed and slightly shorter fixations for Bionic font, but that the number of fixations and fixation placement were not really influenced. After analysis of reading metrics, namely reading speed, fixation duration, number of fixations and fixation placement, it can be concluded that Bionic font does not affect reading significantly, but rather has a slight, negligible effect on some characteristics.

## 8. Further Studies

Additional analysis on the current data could be extended to divide words into letters with Bionic font and letters without, instead of just taking the starting letter. This would give a clearer indication of whether the eye is focused on the Bionic font part of the words or not. Further research could undertake a longitudinal study, allowing participants to enjoy prolonged exposure to Bionic reading in order to determine if, in that instance, reading proficiency will improve. However, a control group would be included that would follow the same intervention but using standard font. As with previous studies [21], the sample can be divided according to reading speed to determine whether the initial reading speed of the participants plays a role in the effectiveness of Bionic font and whether the latter stages of the reading process show marked improvement once the reader is accustomed to the intervention.

The passages used were deliberately chosen from a Hemingway book, since while the story is allegorical in nature, it is considered an easy text to read and follow. Hence, the difficulty of the text was not considered as a factor. In future, different difficulty grades of text can be presented, and a comparative analysis can be conducted to determine if, when reading more difficult texts, bionic reading is a significant influence.

## 9. Conclusions

This study tasked participants with reading two passages from Hemingway’s The Old Man and the Sea, one presented in standard font and the second using Bionic font. Bionic font was designed to improve reading efficiency and train the reader to focus on leading letters in order to more quickly and accurately read the words presented.

The bold letters at the start of the lines did not appear to change the behaviour of the line sweeps, nor did they assist with reading longer, familiar words more efficiently. Hence, in terms of word familiarity and length, Bionic font does not assist the reading process. Low-frequency words were also not more easily read with the addition of Bionic font. The fact that it does not assist with unfamiliar words indicates that the single fixation point required to complete the word is not successful with low-frequency words. Bionic font did result in a minor improvement in terms of lowering fixation durations, but at the same time, there was a minor decrease in reading speed. Paired testing indicated that the use of Bionic reading did not significantly change reading behaviour in terms of reading speed, fixation durations or number of fixations. Hence, it can be concluded that Bionic font is not successful in increasing reading efficiency.

These types of studies, whereby reading interventions or aids are investigated, are beneficial to instructors and publishers in assisting them in determining the best way to publish texts for low-skilled or beginner readers in order to enhance engagement with the text. In particular, it could have an impact on how instructional material is presented in the classrooms, particularly in low-literacy areas. Furthermore, in order to negate the snowball effect of literacy problems at the school level, academic texts or materials could also be presented in formats such as Bionic font if they are shown to be beneficial to understanding and comprehension. Precluding this, a digital format with access to tools that can auto-format the text in a desired manner can be recommended for use for vulnerable demographics.

Limitations of the current study include the makeup of the sample, as it included only staff and students, and the text used. More difficult and a variety of texts would present a more robust study. Furthermore, a longitudinal study might have vastly different results from the single exposure used in the current study.

More in-depth analysis on the breakdown of reading speeds and how that is impacted by Bionic font might be helpful in determining how the font should be implemented. Furthermore, future studies using more difficult texts, academic texts or using a longitudinal intervention to determine whether the long-term effects of Bionic font are not more prominent. Additionally, a demographic that includes at-risk or low-literacy individuals is more conducive to the intervention presented, as these would be the target of such interventions. Overall, the underlying premise of Bionic reading remains intriguing and warrants further investigation as a potential means to overcome reading difficulties.

## Data Availability

Data is available from the author on request.
